# Early protective and risk factors for allergic rhinitis at age 4½ yr

**DOI:** 10.1111/j.1399-3038.2011.01153.x

**Published:** 2011-06

**Authors:** Bernt Alm, Emma Goksör, Hrefna Thengilsdottir, Rolf Pettersson, Per Möllborg, Gunnar Norvenius, Laslo Erdes, Nils Åberg, Göran Wennergren

**Affiliations:** 1 Department of Paediatrics, University of GothenburgGothenburg, Sweden; 2 Paediatric Outpatient Clinic, Central Infant Welfare UnitMölndal, Sweden; 3 Fyrbodal Health Care Region, Central Infant Welfare UnitUddevalla, Sweden; 4 Paediatric Outpatient ClinicSkene, Sweden

**Keywords:** allergic rhinitis, allergic heredity, cohort study, epidemiology, fish

## Abstract

Allergic heredity plays a major role in the development of allergic rhinitis. In addition the introduction of food may influence the risk of subsequent allergic disease. The aim of this study was to analyse early risk factors and protective factors for allergic rhinitis at preschool age. Data were obtained from a prospective, longitudinal study of a cohort of children born in the region of western Sweden in 2003 and 8,176 families (50% of the birth cohort) were randomly selected. The parents answered questionnaires at 6 and 12 months and at 4½ yr of age. The response rate at 4½ yr was 4,496, i.e. 83% of the 5,398 questionnaires distributed at 4½ yr. At 4½ yr of age, 5.5% reported symptoms of allergic rhinitis during the last year. In the multivariate analysis, independent risk factors for allergic rhinitis were: allergic sensitisation to food allergens at 4½ yr (OR 10.21; 95% confidence interval 4.22–24.73), recurrent wheeze at 4½ yr (3.33; 1.56–7.10), doctor-diagnosed eczema at 4½ yr (2.72; 1.62–4.55), parental rhinitis (2.21; 1.39–3.53), eczema first year (1.97; 1.19–3.26) and male gender (1.82; 1.13–2.94). The risk was reduced with fish introduction before 9 months (0.49; 0.29–0.82). In conclusion, we found that previous and present allergic disease, heredity and male gender increased the risk of allergic rhinitis at 4½ yr of age. The introduction of fish before the age of 9 months reduced the risk.

Allergic rhinitis is not a common disease at pre-school age, but at this age symptoms of allergic rhinitis start to appear. The prevalence at 4–5 yr has been reported to vary between 5% (1) and 9.6% (2) and at 6–7 yr between 7.2% (3) and 8.5% (4). Known risk factors include among others allergic heredity, allergic sensitisation and own allergic disease (5, 6). There is controversy concerning the role of parental smoking, male gender, breast-feeding, introduction of food and rural childhood.

There is currently considerable interest in the effects of fish and fish products, especially omega-3 fatty acids, on the development of allergic disease (7). The timing of introduction of solid foods and the exposure to potential allergens in early life has been suggested to influence the maturing immune system (8, 9).

We have previously described a positive influence on the prevalence of eczema following the early introduction of fish (10) and, in a recent review (7), it was found that several epidemiological studies both on maternal fish intake during pregnancy and on fish intake during infancy reported inverse associations between fish intake and atopic outcomes. Intake of fish or omega-3 fatty acids has also been found to have a protective effect on asthma development (11–13). However, the results on allergic rhinitis are not consistent. Some studies report a reduced risk (2, 11), while others find no association (4, 14, 15).

In this paper, the aim was to investigate the prevalence of allergic rhinitis at 4½ yr of age in a western Sweden population and to analyse early risk factors and protective factors for allergic rhinitis at preschool age.

## Methods

Data were obtained from a prospective, longitudinal cohort study of children born in the region of western Sweden in 2003. The region has 1.5 million inhabitants, one sixth of the Swedish population. It comprises urban, rural and coastal areas and the largest city is Gothenburg, with 500,000 inhabitants.

Eight thousand one hundred and seventy-six families (50% of the birth cohort) were randomly selected. After written informed consent was obtained, the parents answered questionnaires at 6 and 12 months and at 4½ yr of age. The response rate at 6 months of age was 69%, while it was 60% at 12 months of age. The response rate at 4½ yr was 4496, i.e. 83% of the 5398 questionnaires distributed. This equals 55.0% of the families that were initially contacted. After supplementation with data from the Swedish Medical Birth Register (MBR), the database consists of 4171 infants with data from all questionnaires and the MBR. Details relating to the questionnaires at 6 and 12 months have been published previously (16, 17). Data on infant feeding including breast-feeding were obtained in the 12-month questionnaire. All variables in the analysis were taken from the questionnaires answered by the parents. The medical records were not reviewed.

The questions relating to allergic rhinitis in the questionnaire were: (i) ‘Has your child experienced sneezing, runny nose, nasal blockage or red and itching eyes after contact with: furry animals, leafing or grass?’; (ii) ‘Has your child experienced these symptoms during the last 12 months?’ and (iii) ‘Has your child been diagnosed with hay fever or allergic rhinitis by a doctor?’

‘Current allergic rhinitis’ was defined as the group of infants whose parents answered yes to (i) and (ii) and/or had been diagnosed by a doctor (iii).

Questions concerning important covariates were:

Allergy testing: ‘Has your child undergone allergy testing (skin prick test and/or blood test)? (Yes/No). If yes: What has the test shown positive reactions for? (Multiple answers are possible): Dog, cat, horse, rabbit, house dust mite, egg, milk, fish, wheat, peanut, soy bean, timothy grass, birch, mugwort, moulds or pea.’ (Asked at 4½ yr of age).

Fish: ‘When did you start the introduction of fish? (Age in weeks)’ (Asked at 12 months of age).

Neonatal antibiotics: ‘Was your baby admitted to a neonatal ward because of problems after birth? (yes/no). If yes, did your baby get penicillin or other antibiotics? (yes/no).’ (Asked at 6 months of age).

Medical treatment during pregnancy: ‘Did you as mother take any medications during pregnancy? (yes/no). If yes, please specify.’ (Asked at 6 months of age).

Eczema at one yr: ‘Has your baby had eczema? (Asked at 12 months of age).

Eczema at 4½ yr: ‘Has your child been diagnosed with eczema by a doctor after 12 months of age?, ‘Has your child had eczema the last 12 months?’ and ‘Have you treated the eczema with cortisone ointment the last 12 months?’ (Asked at 4½ yr of age).

Rural childhood: Responding ‘In the country’ as opposed to ‘City center’, ‘Suburban area’ or ‘Other densely populated area’ to the question ‘Where do you live?’ (Asked at 6 months of age).

In the univariate statistical analysis, 2 × 2 tables with the *χ*^2^ test were used and risks were estimated using the Mantel–Haenszel common odds ratio (OR) estimate. In the multivariate analysis, binary logistic regression was used and OR with 95% confidence intervals (CI) were calculated.

In the multivariate analysis, all significant variables with a p-value < 0.01 were initially included. This cut-off level was chosen to avoid problems with multiple testing. Although not reaching this level, the variable ‘antibiotic treatment first week of life’ (p = 0.032) was included since there is evidence that exposure can lead to an increased risk of subsequent asthma (16, 18). Since effects of breast-feeding and maternal smoking during pregnancy are debated, they too were considered as necessary to analyse and were thus inserted even if they did not reach the significance level of 0.05. Parental education was included in the model to adjust for socioeconomic factors. Rural residence first year was included in the model because of the findings that both rural residence and farm childhood have been associated with a reduced risk of allergic diseases (19, 20).

This resulted in a model containing parental allergic rhinitis, parental eczema, parental asthma, rural residence first year, maternal medication during pregnancy, treatment with broad-spectrum antibiotics first week of life, any bottle feeding first week of life, cat in the home first year, fish introduction before the age of 9 months, fish consumption >1 ×/month first year, recurrent wheeze at 12 months of age, eczema first year, doctor-diagnosed food allergy first year, gender, maternal smoking during pregnancy, parental education, breast-feeding, doctor-diagnosed eczema at 4½ yr, recurrent wheeze at 4½ yr, doctor-diagnosed food allergy at 4½ yr and parental report of positive allergy testing for food or inhalant allergens.

The multivariate analysis was performed with a forward stepwise logistic regression with these variables.

The attributable fraction, AF, was calculated using the formula: 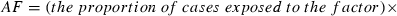

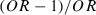
.

The spss v. 17 statistical package was used for statistical calculations.

## Ethical approval

The study was approved by the Ethics Committee, University of Gothenburg.

## Results

At 4½ yr of age, 5.5% (246/4,465) reported symptoms of allergic rhinitis during the last year, i.e. current allergic rhinitis. Of these, 30% also reported their symptoms to be doctor-diagnosed. In the total study population the prevalence of doctor-diagnosed allergic rhinitis with symptoms during the last year, was 1.7% (75/4,465).

**Figure 1:**
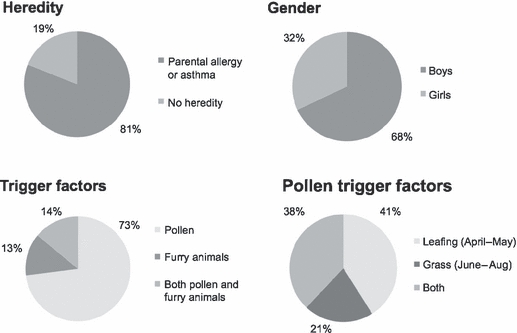
Heredity, gender and trigger factors for rhinitis in the preschool children with current allergic rhinitis.

Of the 246 infants with current allergic rhinitis, 68% were boys and 81% had a positive parental history of allergy or asthma. Seventy-three per cent reported only pollen as a trigger factor, 13% reported that symptoms were triggered by furry animals and 14% by both furry animals and pollen (Fig. 1). Among those who reported pollen as a trigger factor, 41% reported leafing, 21% grass and 38% both as a trigger. Of the children with allergic rhinitis, 40% reported a positive allergy test.

Concurrent allergic manifestations and wheezing at preschool age were common among the children with allergic rhinitis: eczema 37%, food allergy 16% and recurrent wheezing 25%.

The results of the univariate analyses are presented in Table 1. Significant effects with a p-value < 0.05 were found for parental allergic rhinitis, parental eczema, parental asthma, rural residence during the first year of life, maternal medication during pregnancy, antibiotic treatment during the first week of life, any bottle feeding during the first week of life, cat in the home during the first year, fish introduction before the age of 9 months, fish consumption >1 ×/month during the first year, recurrent wheeze at 12 months of age, eczema during the first year, doctor-diagnosed food allergy during the first year, male gender, recurrent wheezing at 4½ yr, doctor-diagnosed food allergy at 4½ yr, doctor-diagnosed eczema at 4½ yr, positive allergy test for inhalant allergens at 4½ yr and positive allergy test for food allergens at 4½ yr.

**Table 1:** Univariate analysis of risk factors for allergic rhinitis with prevalences and ORs

	Allergic rhinitis (*n*/%)						
				
	Yes		No				
					
Variable	n	%	n	%	OR	95% CI	p
Parental allergic rhinitis							
Yes	153	62.4	1662	39.4	2.56	1.96–3.33	<0.001
No	92	37.6	2553	60.6	1	Ref.	Ref.
Parental eczema							
Yes	122	49.8	1567	37.2	1.68	1.30–2.17	<0.001
No	123	50.2	2648	62.8	1	Ref.	Ref.
Parental asthma							
Yes	69	28.2	671	15.9	2.07	1.55–2.77	<0.001
No	176	71.8	3544	84.1	1	Ref.	Ref.
Rural residence first year							
Yes	41	16.9	1019	23.2	0.66	0.47–0.93	0.017
No	202	83.1	3371	76.8	1	Ref.	Ref.
Maternal medication during pregnancy							
Yes	93	38.3	1156	27.6	1.63	1.25–2.13	<0.001
No	150	61.7	3037	72.4	1	Ref.	Ref.
Antibiotic treatment first week of life							
Yes	18	7.3	183	4.4	1.73	1.05–2.86	0.032
No	227	92.7	4016	95.6	1	ref	ref
Any bottle feeding first week of life							
Yes	74	30.2	895	21.3	1.60	1.21–2.12	0.001
No	171	69.8	3305	78.7	1	Ref.	Ref.
Cat in the home first year							
Yes	30	13.0	872	22.1	0.53	0.36–0.78	0.001
No	200	87.0	3074	77.9	1	Ref.	Ref.
Fish introduction before 9 months							
Yes	152	73.8	3040	84.0	0.53	0.39–0.74	<0.001
No	54	26.2	577	16.0	1	Ref.	Ref.
Fish consumption > 1 × /month first year							
Yes	193	84.3	3635	92.6	0.43	0.29–0.62	<0.001
No	36	15.7	291	7.4	1	Ref.	Ref.
Recurrent wheeze at 12 months of age (3 or more episodes)							
Yes	21	9.2	199	5.1	1.87	1.12–2.99	0.009
No	208	90.8	3682	94.9	1	Ref.	Ref.
Eczema first year							
Yes	108	47.2	754	19.2	3.76	2.86–4.93	<0.001
No	121	52.8	3173	80.8	1	Ref.	Ref.
Doctor-diagnosed food allergy first year							
Yes	34	14.8	175	4.5	3.73	2.51–5.54	<0.001
No	195	85.2	3744	95.5	1	Ref.	Ref.
Gender							
Female	75	32.5	1920	48.7	1	Ref.	Ref.
Male	156	67.5	2021	51.3	1.98	1.49–2.62	<0.001
Recurrent wheeze at 4½ yr (3 or more episodes during the last 12 months)							
Yes	61	24.8	184	4.3	7.25	5.24–10.03	<0.001
No	185	75.2	4046	95.7	1	Ref.	Ref.
Doctor-diagnosed food allergy at 4½ yr							
Yes	40	16.3	161	3.8	4.90	3.38–7.12	<0.001
No	206	83.7	4066	96.2	1	Ref.	Ref.
Doctor-diagnosed eczema at 4½ yr							
Yes	91	37.0	583	13.8	3.68	2.80–4.83	<0.001
No	155	63.0	3650	86.2	1	Ref.	Ref.
Positive allergy test (air) at 4½ yr							
Yes	76	42.7	77	2.3	31.22	21.49–45.34	<0.001
No	102	57.3	3226	97.7	1	Ref.	Ref.
Positive allergy test (food) at 4½ yr							
Yes	52	33.8	246	7.1	6.69	4.67–9.57	<0.001
No	102	66.2	3226	92.9	1	Ref.	Ref.

In the univariate analyses of allergic rhinitis, we found no significant association with parity, preterm birth, cohabitation at birth or at 6 months, maternal or paternal disease (other than allergy), maternal education, paternal education, housing, mould or dampness in the house, air pollution, attended maternity care, summer cottage, maternal alcohol use during or after pregnancy, birth weight, admission to neonatal ward, temperament first week, maternal smoking during pregnancy, pacifier use, dog, bird or rodent keeping first year, breast-feeding shorter than 4 months, early introduction of egg, type of spread, type of fat used in cooking, type of fish consumed (lean/fat), vegetarian diet in family, AD-vitamin supplementation or vaccinations.

In order to elucidate whether the small proportion of children not being introduced to fish before 9 months differed from the rest of the population, we performed a stratified analysis. 83.6% of infants had been given fish before 9 months of age, and 16.4% not. Of the infants with a late introduction of fish, 6.6% had a doctor's diagnose of food allergy compared to 3.4% of the early introducers. They also more often had parents with allergy (asthma 21.8% vs. 16.1%, eczema 43.6% vs. 36.2% and rhinitis 46.9% vs. 38.7%, all p < 0.001). However, the effect of early fish introduction was significantly protective in both strata, i.e. there was no significant interaction (all p > 0.3).

In the multivariate analysis, independent risk factors for current allergic rhinitis were: positive food allergy test at 4½ yr, recurrent wheeze at 4½ yr, doctor-diagnosed eczema treated with topical steroids at 4½ yr, parental rhinitis, eczema first year and male gender. The introduction of fish before the age of 9 months reduced the risk (Table 2).

**Table 2:** Independent risk factors for allergic rhinitis at 4½ yr of age in the multivariate analysis, p < 0.05*

Variable	OR	95% CI	p
Positive food allergy test at 4½ yr	10.21	4.22–24.73	<0.001
Recurrent wheeze at 4½ yr	3.33	1.56–7.10	0.002
Doctor-diagnosed eczema treated with topical steroids at 4½ yr	2.72	1.62–4.55	<0.001
Parental rhinitis	2.21	1.39–3.53	<0.001
Eczema first year	1.97	1.19–3.26	0.009
Male gender	1.82	1.13–2.94	0.014
Fish introduction before 9 months	0.49	0.29–0.82	0.007

* Variables in the model were: Parental allergic rhinitis, parental eczema, parental asthma, rural residence first year, maternal medication during pregnancy, treatment with broad-spectrum antibiotics first week of life, any bottle feeding first week of life, cat in the home first year, fish introduction before the age of 9 months, fish consumption > 1 × /month first year, recurrent wheeze at 12 months of age, eczema first year, doctor-diagnosed food allergy first year, gender, maternal smoking during pregnancy, parental education, breast-feeding shorter than 4 months, doctor-diagnosed eczema at 4½ yr, recurrent wheeze at 4½ yr, doctor-diagnosed food allergy at 4½ yr and parental report of positive allergy testing for food or inhalant allergens.

Fish introduction before the age of 9 months was protective against reported positive allergy test (skin prick or blood), both for inhalant (OR 0.50, 95% CI 0.33–0.76) and food allergens (0.59; 0.43–0.82).

The attributable fraction for not introducing fish before 9 months of age was calculated as 10.5% (95% CI 3.4%–15.2%).

## Discussion

The main finding in this study was that positive allergy test, recurrent wheeze, eczema, heredity and male gender increased the risk of allergic rhinitis at 4½ yr of age. The risk was reduced if fish was introduced before the age of 9 months.

Our definition of allergic rhinitis is very similar to the definitions in the ISAAC study (3) and of Kull et al. (2). The studies by Nafstad et al. (11) and Dunder et al. (14) both required a doctor's diagnosis. Although it would be preferable to have doctor-diagnosed allergic rhinitis as the endpoint of the study, we decided to use ‘current allergic rhinitis’ as the endpoint, since the small numbers in the doctor-diagnosed group yield very low power.

The main explanation for the relatively low figure of 40% with positive allergy test is most likely that many children with symptoms had not yet undergone allergy testing. It is also possible that some children with symptoms of allergic rhinitis do not yet test positive at this young age (21). This is why we did not want to limit the analysis to this smaller group.

Allergic rhinitis, and especially doctor-diagnosed allergic rhinitis, is not common at preschool age. In our material, only 75 infants (1.7%) had a doctor-diagnosed allergic rhinitis, while 246 (5.5%) reported symptoms compatible with allergic rhinitis. The prevalence of allergic rhinitis is comparable to that seen in previous studies (1–3, 15). From the Oslo Birth Cohort (1), it is reported that 5.5% of 4 yr olds had received a diagnosis of allergic rhinitis. In the Swedish BAMSE study, the calculated prevalence at age 4 was 9.6% (2). This is somewhat higher than ours, but the difference might be due to the fact that Kull et al. included infants with symptoms during the last 24 months. In the ISAAC study, the Swedish figure (Stockholm) was 5.9% among 6 to 7 yr olds when eye symptoms were included (3). However, in the Manchester Asthma and Allergy Study, the prevalence of rhinitis at 5 yr was higher, current rhinitis 26.1% and rhinoconjunctivitis 12.1%. The prevalence of doctor-diagnosed allergic rhinitis/hay fever was 5.3% (6). Also, an Italian study by Peroni et al. reported a high prevalence of rhinitis in 3–5 yr olds, 16.8%. The study was based on the ISAAC written questionnaire (5).

Not surprisingly, allergic heredity and own allergic disease emerged as the by far strongest risk factors for allergic rhinitis. This emphasises the strong hereditary component in this disease and is in consonance with several previous studies (5, 6). Furthermore, in the present study, a strong link with positive allergy test was seen, particularly with allergic sensitization to food, which exhibited an OR of about 10. A strong link between allergic sensitization and rhinitis in early childhood is consistently found (5, 6, 22). The German MAS study has pointed out sensitization to food allergens during early childhood as a predictor of allergic rhinitis and asthma at age 5 yr (23). Furthermore, allergic rhinitis until the age of 5 yr has recently been reported to be a significant predictor of development of asthma between ages 5 and 13 yr (24).

Like earlier studies, we find that rhinitis at preschool age often occurs concomitantly with wheeze and eczema. In the present study, recurrent wheeze at age 4½ yr was an independent risk factor, as found in also some other studies (5). Already at this young age there seems to be an association between upper and lower airway symptoms. Also the strong association with eczema has been pointed out in other studies (5, 6).

In addition, male gender appeared as an independent risk factor for allergic rhinitis at preschool age. This is not found consistently although it has been seen in some other studies (5).

In consonance with several other studies we did not see any effect of maternal smoking or short breast-feeding (5, 6).

A protective effect of the early introduction of fish on allergic rhinitis at preschool age seen in this study is supported by previous reports of a reduced risk of allergic disease following a high fish intake during pregnancy and during infancy (2, 11, 25).

The composition of fatty acids in the diet (omega-3) and its implications on the developing immune system and the Th1/Th2 balance has been discussed as a possible pathophysiological mechanism. However, it is possible that the effect of fish is mediated not only via omega-3 fatty acids but also by other components of fish. In the present follow-up and in our 1-yr follow-up on eczema (10), we noted that the type of fish ingested did not influence the risk-reducing effect (lean/white or fat/oily). This has also been found in other studies (26, 27).

The timing of introduction of different foods, with a window of opportunity during the first year of life, seems to be of importance for the maturing immune system (8, 9). In addition, the interaction between potential allergens and the immune system in the gut stimulates the development of tolerance and a Th1 balanced immune response (8, 9). Our finding of a protective effect of fish intake in early life is in line with this reasoning. Furthermore, the timing of introduction of fish, being an independent protective factor in our study, seems to be more important than the frequency of fish intake. This indicates that early introduction of solid foods stimulates the development of tolerance and that delayed introduction should not be recommended to avoid subsequent allergic disease (28).

Other possible confounding factors often discussed are parental allergic disease and early allergic manifestations in the child itself. These factors could influence the timing of introduction of solid foods. We have tried to adjust for this difference by controlling for parental rhinitis, asthma and eczema, as well as for wheezing, eczema and doctor-diagnosed food allergy in the child itself. The introduction of fish before the age of 9 months was still an independent protective factor for allergic rhinitis at preschool age. Furthermore, we found no significant interactions, i.e. the effect of early fish introduction was protective irrespective of heredity or own allergic disease.

There is also a possibility that the small proportion of infants with a delayed introduction of fish in some other way can differ from the rest of the population. Therefore, we tested potential confounding variables indicative of life-style in the multivariate model. In doing that, we found no effects of adherence to vaccinations or maternity care, AD-vitamin supplementation, vegetarian life-style and housing. However, even with these adjustments, there is always a risk of residual confounding.

The effects of early fish introduction were similar as those reported earlier for recurrent wheezing at 4½ yr of age (OR 0.6; 0.4–0.8) (29).

The attributable fraction of 10.5% implies that approximately one tenth of the cases with current allergic rhinitis at preschool age could statistically be attributed to the introduction of fish after the age of 9 months.

Another early exposure with possible impact on the maturing immune system is the exposure to a farming environment in early childhood, with a suggested protective effect on allergic disease (19, 20, 30). In the univariate analysis we saw a reduced risk of allergic rhinitis at preschool age in children who lived in a rural residence during the first year of life. However, the association did not reach significance in the multivariate analysis. This might be due to the fact that we did not have data on farming environment, only rural residence. Therefore the data do not allow analysis of the exposure to farming environment exclusively.

### Weaknesses and strengths

A weakness of the study is that the data were collected by postal questionnaires. As a result, the endpoint is not objectively measured. The strengths are the large size of the birth cohort and the good response rate of 70% initially and the current 83% in this follow-up. In all, we have data from all the questionnaires relating to more than four thousand children. Moreover, as reported earlier, the material appears to be largely representative of the population (16).

## Conclusions

Positive allergy test, recurrent wheeze, eczema, heredity and male gender were independent risk factors for allergic rhinitis at age 4½ yr. We also found that the introduction of fish before the age of 9 months reduced the risk of allergic rhinitis at preschool age.
